# Selective Tropism of Dengue Virus for Human Glycoprotein Ib

**DOI:** 10.1038/s41598-018-20914-z

**Published:** 2018-02-09

**Authors:** Nattapol Attatippaholkun, Nont Kosaisawe, Yaowalak U-Pratya, Panthipa Supraditaporn, Chanchao Lorthongpanich, Kovit Pattanapanyasat, Surapol Issaragrisil

**Affiliations:** 1 Siriraj Center of Excellence for Stem Cell Research, Faculty of Medicine Siriraj Hospital, Mahidol University, Bangkok, Thailand; 2 Siriraj Center of Excellence for Flow Cytometry, Faculty of Medicine Siriraj Hospital, Mahidol University, Bangkok, Thailand; 3 Siriraj Laboratory for System Pharmacology, Faculty of Medicine Siriraj Hospital, Mahidol University, Bangkok, Thailand; 4 Division of Hematology, Department of Medicine, Faculty of Medicine Siriraj Hospital, Mahidol University, Bangkok, Thailand; 5 Molecular Medicine Program, Faculty of Science, Mahidol University, Bangkok, Thailand

## Abstract

Since the hemorrhage in severe dengue seems to be primarily related to the defect of the platelet, the possibility that dengue virus (DENV) is selectively tropic for one of its surface receptors was investigated. Flow cytometric data of DENV-infected megakaryocytic cell line superficially expressing human glycoprotein Ib (CD42b) and glycoprotein IIb/IIIa (CD41 and CD41a) were analyzed by our custom-written software in MATLAB. In two-dimensional analyses, intracellular DENV was detected in CD42b^+^, CD41^+^ and CD41a^+^ cells. In three-dimensional analyses, the DENV was exclusively detected in CD42b^+^ cells but not in CD42b^−^ cells regardless of the other expressions. In single-cell virus-protein analyses, the amount of DENV was directly correlated with those of CD42b at the Pearson correlation coefficient of 0.9. Moreover, RT- PCR and apoptosis assays showed that DENV was able to replicate itself and release its new progeny from the infected CD42b^+^ cells and eventually killed those cells. These results provide evidence for the involvement of CD42b in DENV infection.

## Introduction

Dengue infection is the most prevalent arthropod-borne viral disease in subtropical and tropical regions of the world caused by dengue virus (DENV), a single positive-stranded RNA virus. The global burden of DENV infection is large; an estimated 50 million infections per year occur across approximately 100 countries. Thailand is one of the biggest dengue-endemic countries in the world since 1987. Until present, dengue is the leading cause of children hospitalization and its outbreaks continue to pose many deaths every year in Thailand. Generally, dengue infection is an uncomplicated asymptomatic fever called dengue fever. However, in a small proportion, it is life threatening called severe dengue^1^.

Autopsy and clinical findings in humans, as well as studies involving nonhuman primates, have indicated that cells of the mononuclear phagocyte lineage are the primary cell targets, for instance, macrophages and dendritic cells^2,3^. Therefore, many surface molecules utilized by DENV to infect these target cells were identified such as DC-SIGN and mannose receptor^4,5^. However, the death of dengue patients is not caused by the malfunction of the mononuclear phagocyte lineage. Instead, one of the most common causes of death is massive bleeding which is often caused by the malfunction of megakaryocyte-platelet lineage^6–10^. Although previous reports demonstrated that DENV infects the cells in this lineage^11,12^, the platelet receptor that defines the infection has been still unclear^12–14^.

On the plasma membrane of megakaryocyte-platelet lineage, glycoproteins are predominantly located including CD41 (glycoprotein IIb), CD41a (glycoprotein IIb/IIIa) and CD42b (glycoprotein Ib). CD41 associates with CD61 (glycoprotein IIIa) to form a complex CD41a, which functions as the fibrinogen receptor in platelets accelerating platelet aggregation. CD42b is a platelet adhesion receptor, which functions as a component of the glycoprotein Ib-V-IX complex on platelets. The complex binds von Willebrand factor allowing platelet adhesion at sites of vascular injury^15,16^.

Until now, cell-surface molecules, which are of paramount importance for the design to control the severity of severe dengue either dengue hemorrhagic fever or dengue shock syndrome, were not completely unraveled^17^. Research on DENV infection into human host cells to define the tropism of cell-surface molecule, which represents an attractive molecular target to counteract the progression of the disease either by antiviral agents or by immunotherapy, has still presented interesting challenges^18^.

To identify new candidate molecule, which is specific to megakaryocyte-platelet lineage and might be used by DENV for causing massive bleeding in dengue patient, cells superficially expressing human platelet receptors, MEG-01 cells, were used as a model to demonstrate DENV tropism among the receptors. These particular cells naturally express almost any platelet receptors without being genetically engineered^19^. They display their phenotypic properties closely resemble to those of primary megakaryoblasts and are able to produce platelet like particles closely similar to human platelets^20^. They are also susceptible to DENV infection^21^. Therefore, these cells were infected with DENV and its tropism relating to the surface receptors of human platelets was analyzed by flow cytometry.

## Materials and Methods

### Immunostaining

We have published the in-depth staining protocol in ref.^22^. Briefly, anti-DENV complex monoclonal antibody, clone D3-2H2-9-21 (Millipore) was directly conjugated to phycoerythrin (PE) using LYNX Conjugation Kit (AbD Serotec) and kept at 4 °C until used. Cell-surface molecules were stained with the following mouse monoclonal antibodies to human molecules: allophycocyanin (APC)-anti-CD41 (BioLegend) or fluorescein isothiocyanate (FITC)-anti-CD41a (BD Pharmingen) or Peridinin chlorophyll (PerCP)–anti-CD42b (BioLegend®) at 4 °C for 30 minutes. Intracellular DENV was stained at 25 °C after cell surface staining. The cells were washed once with PBS and fixed with 4% paraformaldehyde in PBS for 20 minutes. The fixed cells were washed once with PBS and permeabilized with BD Perm/Wash® buffer (BD Pharmingen) for 20 minutes followed by PE-anti-DENV complex antibody for 1 hour. After incubation with the antibodies, the cells were washed once with PBS and fixed with 1% paraformaldehyde in PBS. The staining was applied to uninfected cells in parallel with DENV-infected cells.

### Two-dimensional flow cytometric analysis

The immunostained cells (1 × 10^5^ cells) were analyzed by either FACSCalibur or BD LSR II (Becton Dickinson). Flow cytometry standard data files were analyzed in two-dimension using CellQuest (Becton Dickinson) or FlowJo. Live cells were gated on Forward scatter and Side scatter double dot. Unstained cells were used to determine the compensation and cut-off cells without surface molecules of interest. Uninfected cells were used to determine the compensation and cut-off cells without DENV.

### Three-dimensional flow cytometric analysis

Flow cytometry standard data files were analyzed in three-dimension using custom-written software in MATLAB. The files were read using FCS data reader. Forward scatter and Side scatter were plotted in two-dimension using scatplot(x,y,’circles’). Live cells were gated in the plot using impoly(gca). Gated positions were stored using inpolygon(xq,yq,xv,yv). The fluorescence intensity of each gated cell were plotted in three-dimension using scatter(x,y,a,c,’filled’). The intensity of DENV-PE were displayed as heatmap using caxis([cmin,cmax]).

### Single-cell virus-protein analysis

Pearson correlation coefficients and their *P*-values between the fluorescence intensities of intracellular DENV and surface glycoprotein were calculated in MATLAB using [RHO,PVAL] = corr(x,y,’type’,’pearson’). Regression lines were plotted using plotregression(x, y). x and y were the fluorescence intensities of each single cell. The coefficients were statistically compared using compare_correlation_coefficients(r1, r2, n1, n2). r1 and r2 were Pearson correlation coefficients of interest. n1 and n2 were sample sizes of each coefficient.

### Fluorescence microscopic analysis

After double immunostaining, the cells were centrifuged at 2,000 rpm for 5 minutes. The supernatant was discarded. The cells were mixed with the remaining supernatant at the bottom of the tube. The cells (10 µl) were dropped onto a microscopic slide with its cover slip. The slides were visualized under a fluorescence microscope using the B-2E/C filter for FITC and G-2A filter for PE. The cell pictures were captured and merged using NIS Element D4.10.00 software.

### Cell culture

MEG-01 cells were purchased from the American Type Culture Collection (ATCC) and cultivated in RPMI 1640 (GIBCO) supplemented with 10% FBS (GIBCO) and 2 mM L-glutamine at 37 °C in a 5% CO_2_ humidified atmosphere. Cell numbers were maintained below approximate density of 10^6^ cells/ml.

### Dengue virus infection

MEG-01 cells were adjusted to 3 × 10^5^ cells and spun down. The infected cells were resuspended with 1 ml of DENV at a MOI of 0.5. The uninfected cells were resuspended with MEM containing 2% FBS and 2 mM L-glutamine. All the cultures were mixed and incubated at 37 °C, 5% CO_2_ for 2 hours. The cells were washed twice with PBS and were maintained in fresh medium before being analyzed.

### Dengue virus production

Vero cells were maintained at a concentration of 1 × 10^6^ cells/plate in Minimum Essential Medium (MEM) (GIBCO) containing 10% FBS and 2 mM L-glutamine at 37 OC, 5% CO_2_. DENV type 2 (strain 16681) was added to 80–90% confluent of the cells at a multiplicity of infection (MOI) of 0.1 and incubated at 25 °C with rocking for 2 hours. After incubation, the culture media was replaced with fresh MEM containing 2% FBS and cultured at 37 °C, 5% CO_2_. The supernatant of DENV-infected Vero cells was collected and replaced at 3 and 7 days post infection. The collected medium was centrifuged at 1500 rpm, 4 °C for 5 minutes. The supernatant was aliquoted and stored at −80 °C until used.

### RNA extraction, RT-PCR and gel electrophoresis analysis

Dengue viral RNA was extracted from 50 µl of the culture supernatant using a QIAamp viral RNA mini kit (Qiagen). The RNA was reverse transcribed and amplified using the nested RT-PCR as previously described^23^. The nucleotide sequences of outer primers including forward and reverse primers for dengue viral E gene were 5′TGGCTGGTGCACAGACAATGGTT3′ and 5′GCTGTGTCACCCAGAATGGCCAT3′, respectively. The nucleotide sequences of inner primers including forward and reverse specific for DENV type 2 E gene were 5′ATCCAGATGTCATCAGGAAAC3′ and 5′CCGGCTCTACTCCTATGATG3′, respectively. The RT-PCR products (346 base pairs) were analyzed by electrophoresis on a 2% agarose gel stained with RedSafe (iNtRON Biotechnology).

### Apoptosis assay

Uninfected and infected cells (1 × 10^5^ cells) were labeled following the kit protocol using FITC Annexin V Apoptosis Detection Kit I (BD Pharmingen). The percentages of apoptotic cells were analyzed by flow cytometry.

## Results

### Tropism of CD42b, CD41 and CD41a in dengue virus by two-dimensional analysis

**Figure 1:**
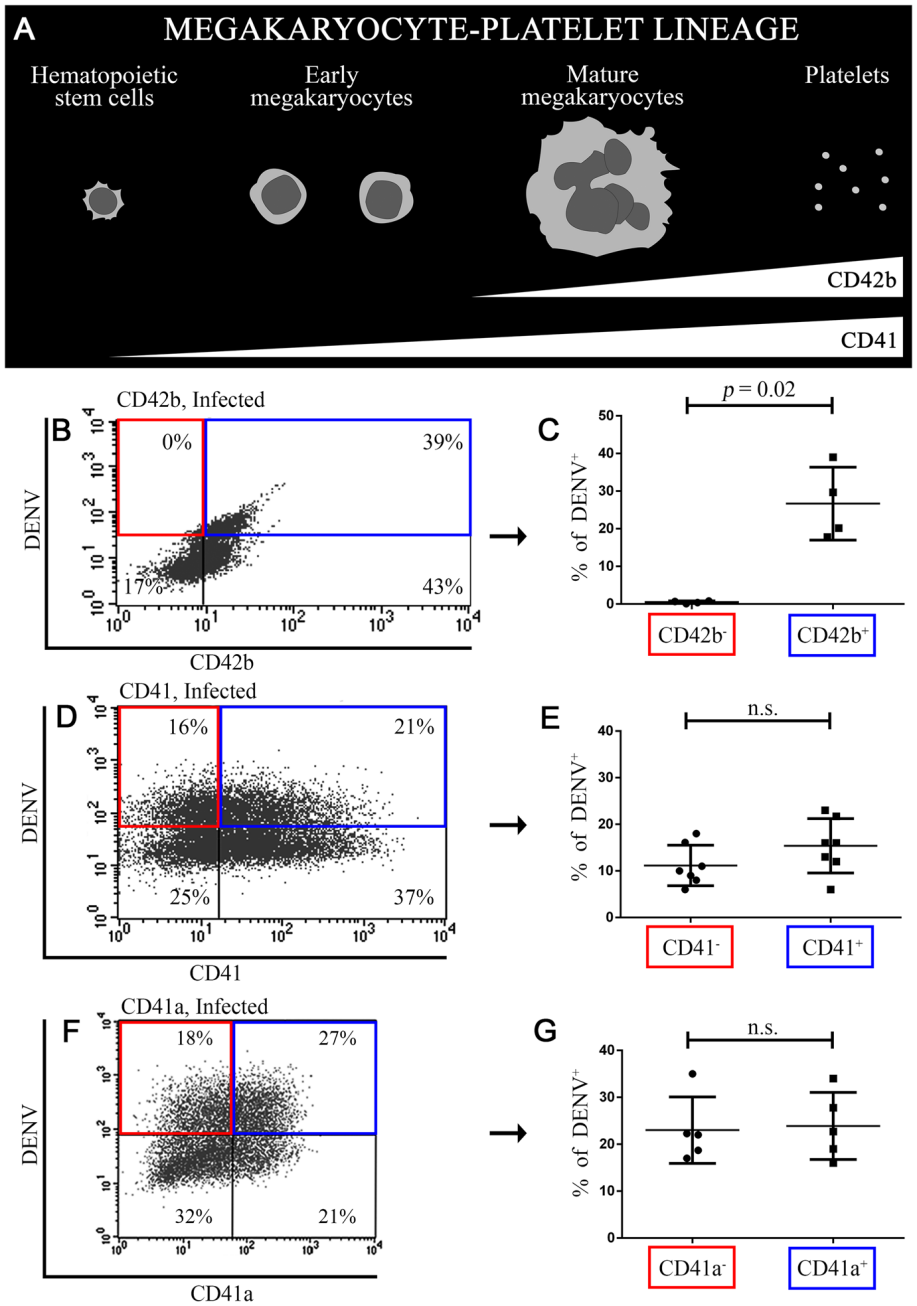
Tropism of CD42b, CD41 and CD41a in dengue virus. (**A**) CD42b is the marker of mature megakaryocytes and platelets^25^. CD41 is the marker of early megakaryocytes^15^. (**B**–**G**) MEG-01 cells were infected with DENV (MOI = 0.5) for 2 hours and washed with PBS. The cultures were maintained in fresh medium for another 7 days before being double immunostained. **(B,D,F) **Representative two-dimensional plot of surface platelet receptors and intracellular DENV (**(B)** CD42b, **(D)** CD41,  **(F)** CD41a). **(C,E,G)** Percentage of DENV^+^ cells without and with expressing platelet receptors (**(C)** CD42b (n = 4),  **(E)** CD41 (n = 7), **(G)** CD41a (n = 5)). Error bars represent mean ± SD. Statistical significance was determined by using the two-tailed Mann–Whitney test.

Rapid depletion of platelet is one of the most common manifestations in dengue patient. Platelet is the late-stage of megakaryocyte-platelet development. Therefore, we firstly investigated the late-stage differentiation marker, CD42b. This marker starts being expressed from mature megakaryocytes to platelets^24,25^ (Fig. 1A). We double immunostained DENV-infected cells with CD42b-PerCP and DENV-PE. The processes how to define the quadrant were demonstrated in Fig. S1. Two-dimensional analysis showed that intracellular DENV were detected in CD42b^+^ cells (39%). Surprisingly, intracellular DENV were not detected in CD42b^−^ cells (<1%) (Fig. 1B). When comparing the percentage of DENV^+^ cells between the subpopulations, DENV^+^ cells were detected in CD42b^+^ cells significantly higher than in CD42b^−^ cells (Fig. 1C; *p* = 0.02 in a Mann-Whitney test). DENV infected CD42b^+^ cells but did not infect CD42b^−^ cells suggesting selective tropism of DENV for CD42b. (Fig. 1B,C).

We next investigated DENV tropism of the early-stage differentiation marker, CD41. This marker starts being expressed in hematopoietic stem cells and prominently expressed in early megakaryocytes^15^ (Fig. 1A). We double immunostained DENV-infected cells with CD41-APC and DENV-PE. Two-dimensional analysis showed that intracellular DENV were detected in 21% of CD41^+^ cells. However, intracellular DENV were also detected in 16% of CD41^−^ cells (Fig. 1D). The percentage of DENV^+^ cells was not different in CD41^+^ cells as compared with CD41^−^ cells (Fig. 1E; *p* = 0.21 in a Mann Whitney test). DENV infected both CD41^+^ and CD41^−^ cells suggesting negligible tropism of DENV for CD41 (Fig. 1D,E). DENV tropism of CD41a was also investigated. We double immunostained DENV-infected cells with CD41a-FITC and DENV-PE. The stained cells were demonstrated under the fluorescence microscope herein to confirm that our immunostaining method detected platelet receptors superficially and DENV intracellularly^22^ (Fig. S2). Similarly, the analysis showed that intracellular DENV were detected in both CD41a^+^ cells (27.8%) and CD41a^−^ cells (18.7%) (Fig. 1F). The percentage of DENV^+^ cells was also not different in CD41a^+^ cells as compared with CD41a^−^ cells (Fig. 1G; *p* = 0.80 in a Mann Whitney test). Therefore, the tropism of DENV for CD41a (Fig. 1F,G) was also similar to CD41 (Fig. 1D,E).

### Specificity of CD42b, CD41 and CD41a in dengue virus infection by two-dimensional analysis

**Figure 2:**
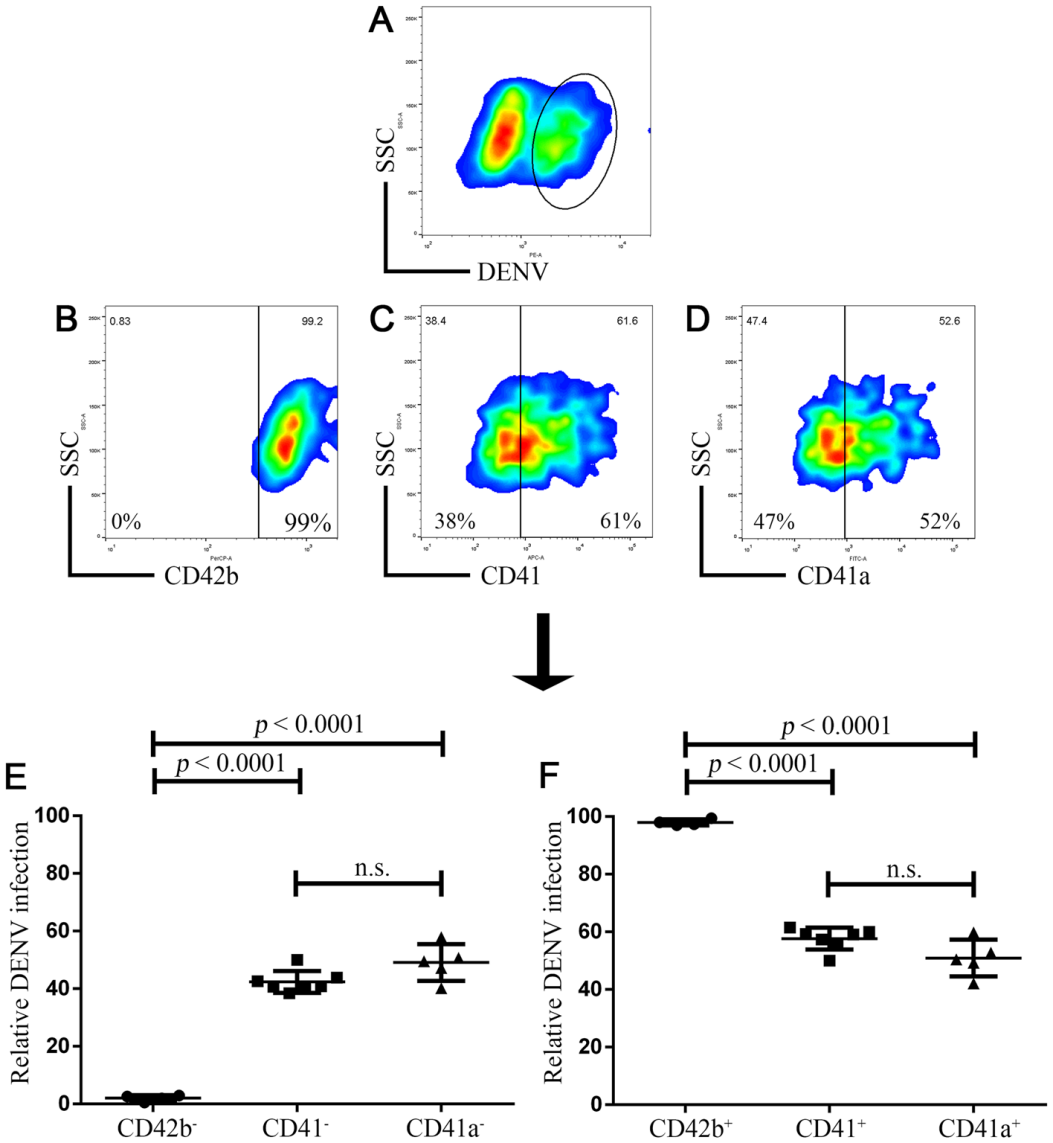
Specificity of CD42b, CD41 and CD41a in dengue virus infection. The cells from Fig. 1 were further analyzed. (**A**) Intracellular DENV^+^ cells were firstly gated (black eclipse) and characterized with surface platelet receptors ((**B**) CD42b, (**C**) CD41, (**D**) CD41a). The percentage of DENV^+^ cells after this gating was represented as a relative DENV infection. (**E**) The relative DENV infection in cells without expressing CD42b (n = 4), CD41 (n = 7) and CD41a (n = 5). (**F**) The relative DENV infection in cells with expressing CD42b (n = 4), CD41 (n = 7) and CD41a (n = 5). Error bars represent mean ± SD. Statistical significance was determined by using one-way ANOVA test.

We extended these observations by gating only intracellular DENV^+^ cells (Fig. 2A) and characterizing the cells with the surface platelet receptors of CD42b, CD41, and CD41a (Fig. 2B–D). The percentages of DENV^+^ cells after this gating were shown as a relative DENV infection (Fig. 2). The cutting points of cells with or without expressing the receptors were based on the no-stained cells (Fig. S3). The specificity of CD42b in DENV infection was marked by an All or None pattern. All of intracellular DENV^+^ cells (99%) were CD42b^+^ cells. None of those cells (0%) were CD42b^−^ cells (Fig. 2B). However, the fraction of intracellular DENV^+^ cells associated with CD41 was negligible. The 38% of those cells were CD41^−^ cells and 61% of those cells were CD41^+^ cells (Fig. 2C). A similar trend was also noted in the case of CD41a. The 47% of those cells were CD41a^−^ cells and 52% of those cells were CD41a^+^ cells (Fig. 2D).

Indeed, when we particularly compared the percentage of intracellular DENV^+^ cells among CD42b^−^, CD41^−^ and CD41a^−^ cells (Fig. 2E), DENV were able to infect average 42% of CD41^−^ and average 49% of CD41a^−^ cells which were significantly higher than CD42b^−^ cells (1% on average) (*p* < 0.0001 in a one-way ANOVA test). DENV did not infect cell that did not express CD42b. This comparison suggested the specificity of CD42b in DENV rather than CD41 and CD41a. However, the comparison among CD42b^+^, CD41^+^ and CD41a^+^ cells still showed that DENV were able to infect all of these cells (Fig. 2E; 99%, 57% and 50% on average, respectively). Even though, the percentage of DENV^+^CD42b^+^ cells was significantly higher than those of the DENV^+^CD41^+^ and DENV^+^CD41a^+^ cells (*p* < 0.0001 in a one-way ANOVA test), this comparison only suggested the difference of specificity between CD42b, CD41 and CD41a. It did not exclude the specificity of CD41 and CD41a out of DENV (Fig. 2E; 57% and 50% on average, respectively).

### Requirement of CD42b in dengue virus infection by three-dimensional analysis

**Figure 3:**
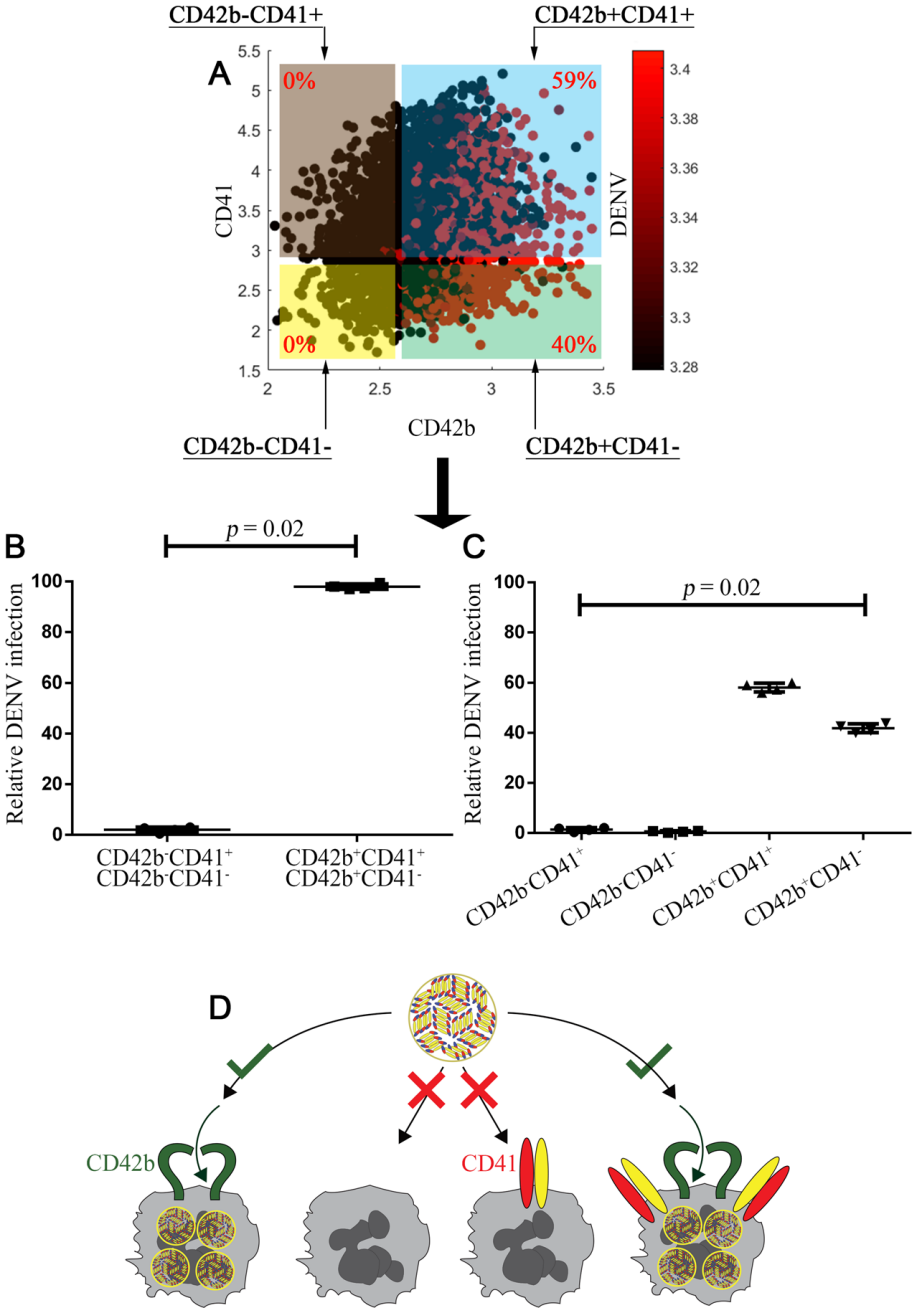
Requirement of CD42b in dengue virus infection. MEG-01 cells were infected with DENV (MOI = 0.5) for 2 hours and washed with PBS. The cultures were maintained in fresh medium for another 7 days before being triple immunostained. Flow cytometry standard data files were analyzed in three-dimension using custom-written software in MATLAB. All the fluorescence intensities were shown as log10 of the actual data. (**A**) Representative three-dimensional plot of surface CD42b, surface CD41 and intracellular DENV. (**B**) The relative DENV infection in CD42b^−^CD41^−/+^ and CD42b^+^CD41^−/+^ subpopulation (n = 4). (**C**) The relative DENV infection in CD42b^−^CD41^+^, CD42b^−^CD41^−^, CD42b^+^CD41^+^ and CD42b^+^CD41^−^ subpopulation (n = 4). Error bars represent mean ± SD. Statistical significance was determined by using the two-tailed Mann–Whitney test. (**D**) Proposed model for the requirement of CD42b in dengue virus infection.

To specify the specificity of DENV infection for CD42b, we triple immunostained DENV-infected cells with CD42b-PerCP, CD41-APC and DENV-PE. The stained cells were firstly analyzed in two-dimension (Fig. S4) and further demonstrated in three dimension (Fig. 3). The present study was the first report to write a custom software in MATLAB to simultaneously demonstrated CD42b, CD41 and DENV in three-dimension. We assumed the similar trend of this analysis with CD41 to the case of CD41a because the tropism and specificity of CD41 and CD41a were similar (Figs 1 and 2). We firstly divided intracellular DENV^+^ cells into 2 subpopulations based on the expression level of CD42b. Three-dimensional analysis showed that DENV were not detected in CD42b^−^ cells regardless of CD41 expression (Fig. 3A; <1% of red dot in brown and yellow square). Strikingly, DENV were exclusively detected in CD42b^+^ cells regardless of CD41 expression (Fig. 3A; 99% of red dot in blue and green square). As comparing the percentage of intracellular DENV^+^ cells between these subpopulations, DENV were detected in CD42b^+^CD41^+/−^ cells significantly higher than in CD42b^−^CD41^+/−^ cells (Fig. 3B; *p* = 0.02 in a Mann-Whitney test). These results specify the specificity of CD42b in DENV.

We further hypothesized that CD42b alone was a key requirement for DENV infection because we could not associate intracellular DENV^+^ cells with CD42b^−^ cells (Figs 1B,C and 2B,E; <1% on average). We divided intracellular DENV^+^ cells into 4 subpopulations based on both CD42b and CD41 (Fig. 3C). The requirement of CD41 for DENV infection was excluded herein because average <1% of intracellular DENV^+^ cells were CD41^+^CD42b^−^. DENV did not infect CD41^+^ cells that did not co-express CD42b. A similar trend was also noted in the case of CD42b^−^CD41^−^ cells. By contrast, the requirement of CD42b were specified herein since average 58% of intracellular DENV^+^ cells were CD41^+^CD42b^+^. CD41^+^ cells must have co-expressed CD42b to be susceptible to DENV. Moreover, intracellular DENV were detected in CD42b^+^CD41^−^ cells but were not detected in CD42b^−^CD41^+^ cells (Fig. 3C; *p* = 0.02 in a Mann-Whitney test). Cells expressing CD42b alone without CD41 were still susceptible to DENV. These results implicate that CD42b alone is the key target for DENV infection (Fig. 3D).

### Dependence of dengue virus infection on CD42b by single-cell virus-protein analysis

**Figure 4:**
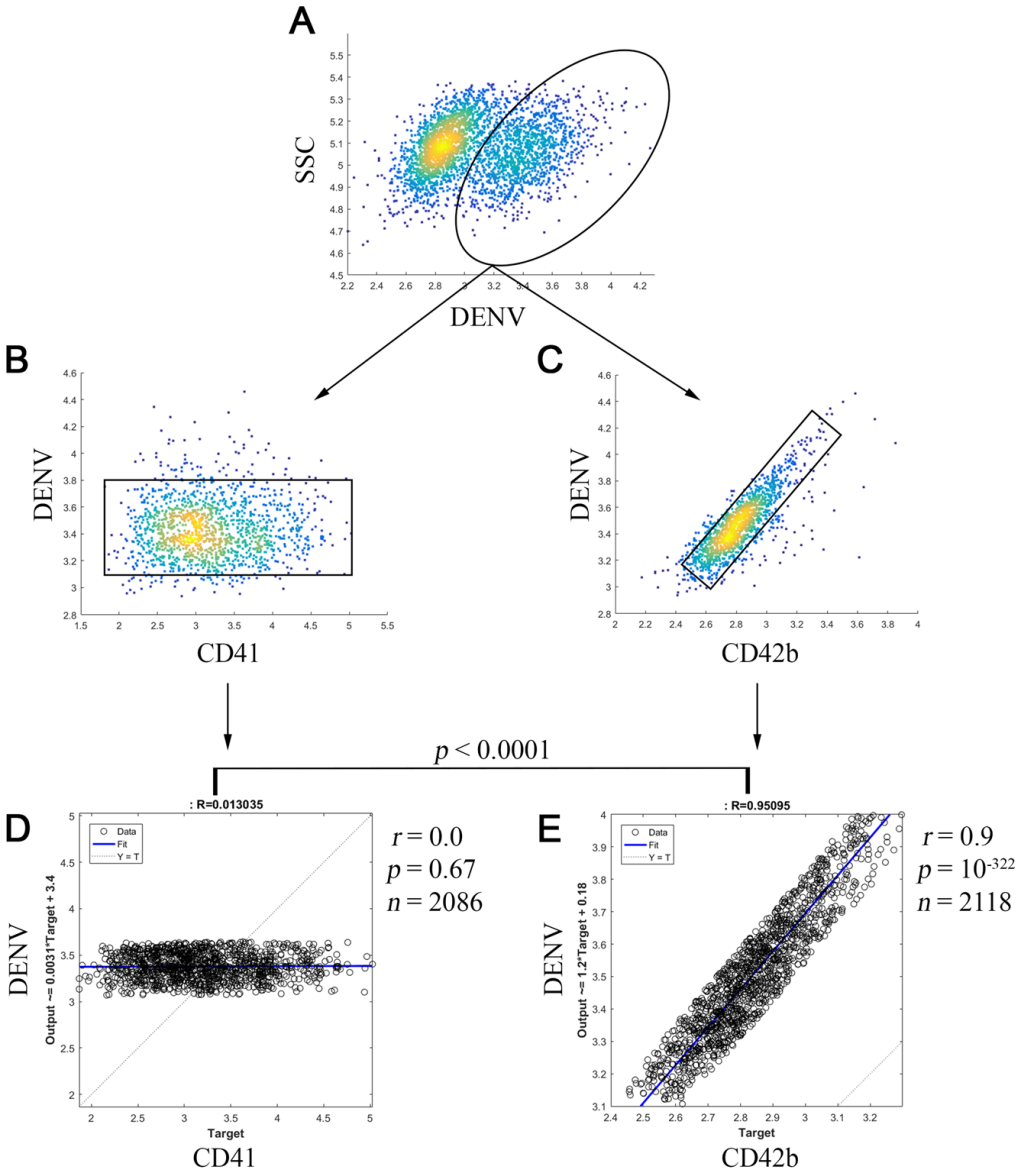
Dependence of dengue virus infection on CD42b. The cells from Fig. 3 were further analyzed. Intracellular DENV^+^ cells were firstly gated ((**A**) black eclipse) and then plotted in two-dimension of intracellular DENV and surface platelet receptors ((**B**) CD41, (**C**) CD42b). The cells were further gated to exclude the non-specific signal (black square). (**D**) Single-cell virus-protein analysis of intracellular DENV and surface CD41 (**E**) Single-cell virus-protein analysis of intracellular DENV and surface CD42b. *r* = Pearson correlation coefficient. *p* = p-value. *n* = the number of analyzed cells. Statistical significance was determined by using Fisher transformation test.

We next hypothesized that DENV infection might depend on CD42b because of the specificity and the requirement (Figs 2 and 3). To investigate its dependency, we triple immunostained DENV-infected cells with CD42b-PerCP, CD41-APC and DENV-PE. We gated intracellular DENV^+^ cells (Fig. 4A) and plotted the fluorescence intensities of DENV with those of CD41 (Fig. 4B) and with those of CD42b (Fig. 4C). We excluded the non-specific signal (Fig. S5) by gating the cells based on those regression lines of each plot (Fig. 4B,C; black square). Each single cell from the gating was individually measured its linear association of the fluorescence intensities. The Pearson correlation coefficient between intracellular DENV and surface CD41 as shown the value of 0 suggested that the amount of intracellular DENV was not correlated with those of surface CD41. (Fig. 4D; *p* = 0.67, *n* = 2086). However, the coefficient between intracellular DENV and surface CD42b as shown the value of 0.9 suggested that the amount of intracellular DENV was directly correlated with those of surface CD42b. (Fig. 4E; *p* = 10^−322^, *n* = 2118). Also, the statistical comparison between these coefficients indicated that those of DENV and CD42b were significantly different from those of DENV and CD41 (Fig. 4E; *p* < 0.0001 in Fisher transformation). These results demonstrate the direct dependence of dengue virus infection on CD42b.

### Dengue virus replicates itself in CD42b^+^ cells

**Figure 5:**
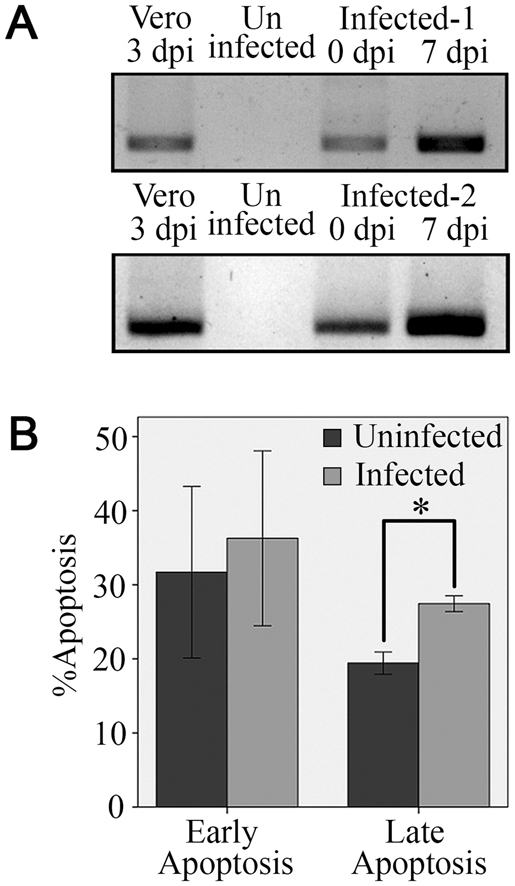
Dengue virus replicates itself in CD42b^+^ cells and eventually kills those cells. MEG-01 cells were infected with DENV (MOI = 0.5) for 2 hours and washed with PBS. The cultures were maintained in fresh medium before performing RT-PCR and apoptosis assays. (**A**) RT-PCR of the culture medium of DENV-infected Vero cells, the culture media of uninfected and DENV-infected MEG-01 cells. dpi = days post infection. Infected-1 and -2 experiments were 2 independent experiments. (**B**) Apoptosis assays of uninfected and DENV-infected MEG-01 cells at 7 dpi. Early apoptosis means Annexin V^+^ PI^−^ cells and late apoptosis means Annexin V^+^ PI^+^ cells. Data are presented as mean ± SEM of four independent experiments. The Mann-Whitney U test was used to assess the significance of differences between the observed data. *p < 0.05.

We further studied on the replication and apoptosis induction capability of dengue virus in CD42b^+^ cells. In this study, those capabilities were the consequence of DENV infection in the CD42b^+^ cells because the virus specifically infected the CD42b^+^ cells. To determine the ability of DENV replication in CD42b^+^ cells, the presence of viral RNA in the culture medium was extracted and amplified using RT-PCR technique. Gel electrophoresis analysis of the RT-PCR products showed that the viral RNA in the culture medium of 7 days post infection (dpi) increased as compared with those of 0 dpi (Fig. 5A). These analyses indicated that DENV was able to replicate itself in CD42b^+^ cells and release its new progenies from those cells.

### Dengue virus kills CD42b^+^ cells

To determine the ability of DENV to attack CD42b^+^ cells, DENV-infected and uninfected cells were stained with FITC-Annexin V and propidium iodide (PI) at 7 dpi. Flow cytometric analysis showed that DENV were able to induce both early apoptosis (Annexin V^+^ PI^−^) and late apoptosis (Annexin V^+^ PI^+^) (Fig. 5B). Comparing with the uninfected cells, the late apoptosis of DENV-infected cells was significantly increased (*p* < 0.05). These assays suggested that the DENV attacks CD42b^+^ cells leading to cell apoptosis.

## Discussion

CD42b, CD41 and CD41a have been shown as core surface markers of megakaryocytes to determine the maturation in stem cell biology^16^ (Fig. 1A). Among the three, we found that CD42b is essential for DENV infection (Fig. 3D). Surprisingly, CD41 and CD41a are dispensable. In addition, we identified the expression level of surface CD42b as also an essential factor. Its level could predict the amount of intracellular DENV (Fig. 4E).

**Figure 6:**
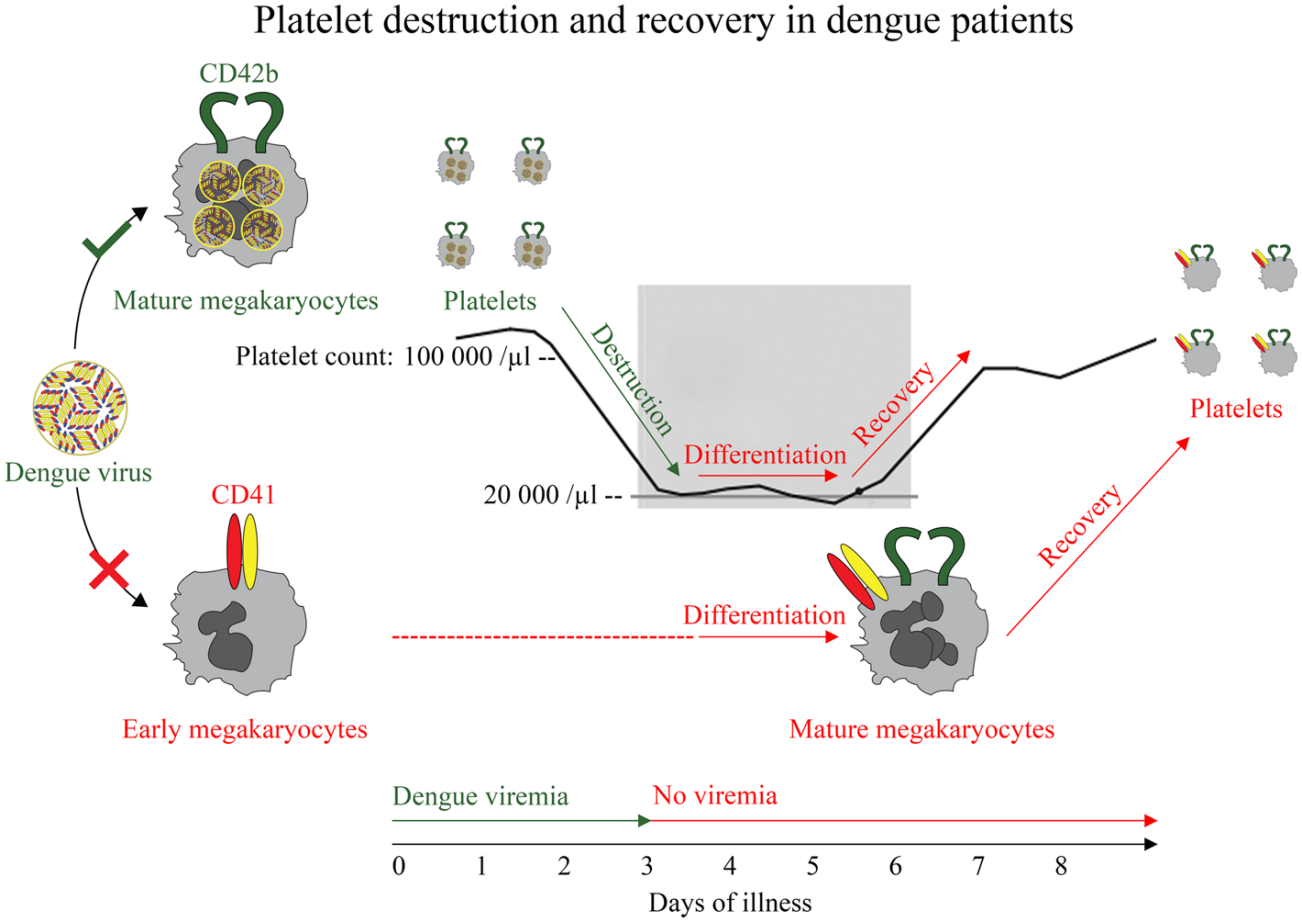
Proposed pathogenesis of platelet destruction and recovery in dengue patients. DENV might attack CD42b^+^ platelets and CD42b^+^ mature megakaryocytes leading to the rapid destruction of platelets. DENV could not attack CD42b^−^ early megakaryocytes explaining the characteristic delay of platelet recovery during the differentiation from early to mature megakaryocytes for 3 days. All new CD42b^+^ megakaryocytes could start producing platelets resulted in the rapid recovery of platelets.

CD42b defining DENV infection could explain its pathogenesis of platelet destruction and recovery in dengue patients as our new hypothesis proposed in Fig. 6. First, platelets rapidly decrease starting at day 2 of the illness. DENV might kill CD42b^+^ mature megakaryocytes and platelets leading to thrombocytopenic state (Fig. 5B). Next, thrombocytopenic state is maintained for 3 days. DENV could not attack CD41^+^CD42b^−^ early megakaryocytes because these cells lack of CD42b expression (Fig. 3D). Since dengue viremia disappears after days 3 of the illness^1^, these early cells would still survive and continue differentiating into CD42b^+^ mature megakaryocytes. The differentiation of early to mature megakaryocytes would take 3 days^15^. Consequently, new mature megakaryocytes could start producing platelets back to blood circulation leading to the rapid increase in platelet count starting at days 6 of the illness.

**Figure 7:**
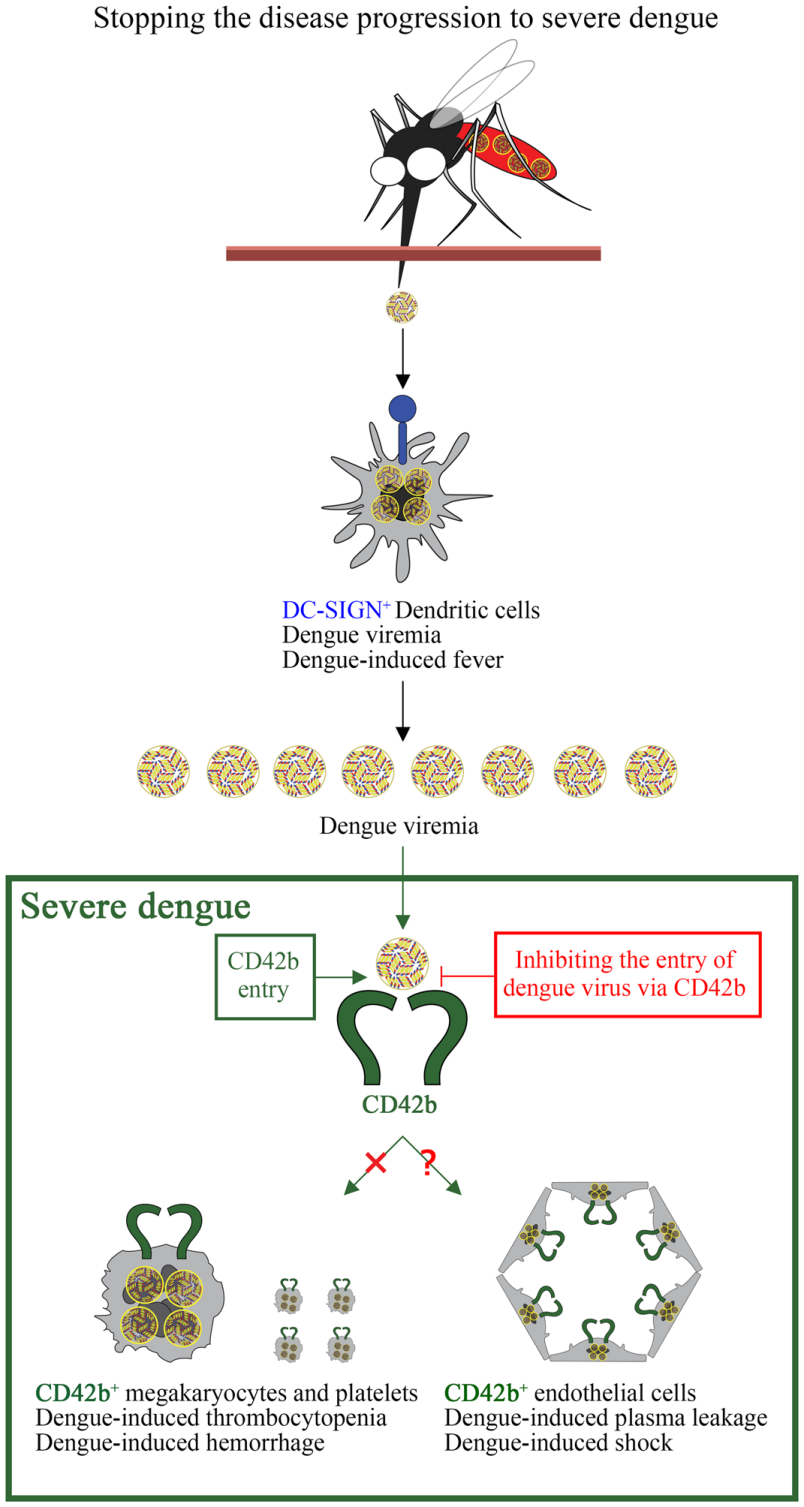
Proposed the strategy to stop the disease progression to severe dengue. *Aedes aegypti* inject DENV into human skin. Dendritic cells phagocytose and amplify DENV leading to viremia causing fever^4,38,39^. DENV in bloodstream may infect CD42b^+^ mature megakaryocytes and platelets leading to thrombocytopenia causing hemorrhage. DENV may also infect CD42b^+^ endothelial cells leading to plasma leakage causing shock. Inhibiting the binding of DENV to CD42b would be able to stop the disease progression from dengue fever to severe dengue.

DENV-induced hypovolemic shock is also well known as the lethal pathogenesis of DENV infection similar to DENV-induced hemorrhage. The hemorrhage is the pathology in platelets^6–11^. However, the shock is the pathology in endothelial cells^26–28^. Interestingly, not only platelets but also endothelial cells superficially express CD42b^29,30^. DENV may attack endothelial cells by targeting CD42b as well. It would explain the dual mode of viral attack to both platelet and endothelial cells via CD42b causing the characteristic of severe dengue, hemorrhage and shock (Fig. 7).

Clinical manifestations of CD42b deficiency patients (Bernard-Soulier syndrome) is interestingly similar to those of thrombocytopenic state in dengue patients including petechial rash and mild to severe thrombocytopenia with a small number of giant platelets on a peripheral blood smear^31,32^. Moreover, the management of both diseases is also similar. Platelet transfusion is not essential even though the patient platelet count is lower than 20,000/mm^3^. The patients need the transfusion only in a critical situation for instance, major surgery and severe trauma^31,33^. DENV might interfere surface CD42b of platelets without reducing its expression since DENV-infected cells still continued expressing CD42b from our results (Fig. 4C). The autoantibodies to the patient platelets induced by DENV might play a crucial role to conceal the CD42b mimicking the deficiency of CD42b^33^. However, CD42b deficiency patients do not manifest plasma leakage leading to hypovolemic shock^31^. DENV may infect endothelial cells through CD42b but the further molecular mechanism causing plasma leakage would not involve the surface CD42b.

Severity of DENV infection varies among the patients from asymptomatic to lethality^1^. Until now, no biomarker is used in routine clinic to predict the severity at the day of diagnosis. Since dengue patients are admitted excessively in hospitals of Thailand^34^, many of them are routinely discharged based on the decreasing of fever and the increasing of platelet counts^35^. However, the increasing could delay for 3 days leading to patient overload^1^ (Fig. 6). To decrease the patient admission, the novel biomarker that could predict the severity at the day of diagnosis would help clinicians to decide whether the patient should be admitted or not. In this study, DENV infected and replicated more effectively in CD42b^high^ cells as compared with CD42b^low^ cells (Fig. 4C). Severity of DENV infection may depend on the expression level of CD42b on surface of its target cells. Platelets and endothelial cells of severe dengue patients might express higher level of CD42b than those of the asymptomatic patients. Therefore, the expression level of CD42b on platelets and endothelial cells might predict dengue severity.

The discovery of the essential in surface CD42b for DENV infection suggests one possible strategy of DENV to attack the cells: by using its surface E protein binding to surface CD42b of its target cells. DENV could not bind to cells without superficially expressing CD42b leading to the incapability of infection (Fig. 3D). X-ray crystallography strategy of the complex between CD42b and E protein would demonstrate the binding site between these proteins. The information from the crystal structure would suggest a strategy for interfering with the binding, related to a successful approach in developing a HIV antiviral compound^36^. Small molecule targeting gp120-binding domain of CD4 inhibits HIV entry by disruption of gp120 and CD4 interaction^37^. Using the small molecule targeting E protein-binding domain of CD42b, it could not stop dendritic cells to phagocytose DENV via DC-SIGN causing dengue fever^4,38,39^. But, it would be able to stop the disease progression from dengue fever to severe dengue including dengue-induced hemorrhage and shock by inhibiting DENV entry to CD42b^+^ megakaryocytes, platelets and endothelial cells (Fig. 7).

## Electronic supplementary material

Supplementary Information
