# Publisher Correction: Selective Tropism of Dengue Virus for Human Glycoprotein Ib

**DOI:** 10.1038/s41598-018-23724-5

**Published:** 2018-04-12

**Authors:** Nattapol Attatippaholkun, Nont Kosaisawe, Yaowalak U-Pratya, Panthipa Supraditaporn, Chanchao Lorthongpanich, Kovit Pattanapanyasat, Surapol Issaragrisil

**Affiliations:** 1 Siriraj Center of Excellence for Stem Cell Research, Faculty of Medicine Siriraj Hospital, Mahidol University, Bangkok, Thailand; 2 Siriraj Center of Excellence for Flow Cytometry, Faculty of Medicine Siriraj Hospital, Mahidol University, Bangkok, Thailand; 3 Siriraj Laboratory for System Pharmacology, Faculty of Medicine Siriraj Hospital, Mahidol University, Bangkok, Thailand; 4 Division of Hematology, Department of Medicine, Faculty of Medicine Siriraj Hospital, Mahidol University, Bangkok, Thailand; 5 Molecular Medicine Program, Faculty of Science, Mahidol University, Bangkok, Thailand

Correction to: *Scientific Reports* 10.1038/s41598-018-20914-z, published online 09 February 2018

The original version of this Article contained typographical errors.

In the Results section, the subheading,

“Tropism of CD42b, CD41a and CD41a in dengue virus by two-dimensional analysis”

now reads:

“Tropism of CD42b, CD41 and CD41a in dengue virus by two-dimensional analysis”

In the Results section, under the subheading ‘Tropism of CD42b, CD41 and CD41a in dengue virus by two-dimensional analysis’,

“The stained cells were demonstrated under the fluorescence microscope herein to confirm that our immunostaining method detected platelet receptors superficially and DENV intracellularly (Fig. S2).”

now reads:

“The stained cells were demonstrated under the fluorescence microscope herein to confirm that our immunostaining method detected platelet receptors superficially and DENV intracellularly^22^ (Fig. S2).”

In the Results section, under the subheading ‘Requirement of CD42b in dengue virus infection by three-dimensional analysis’,

“We further hypothesized that CD42b alone was a key requirement for DENV infection because we could not associate intracellular DENV+ cells with CD42b− cells (Figs 1B and 2B,E; <1% on average).”

now reads:

“We further hypothesized that CD42b alone was a key requirement for DENV infection because we could not associate intracellular DENV+ cells with CD42b− cells (Figs 1B,C and 2B,E; <1% on average).”

In the Results section, under the subheading ‘Dependence of dengue virus infection on CD42b by single-cell virus-protein analysis’,

“(Fig. 4E; *p* = 10^−3^, *n* = 2118).”

now reads:

“(Fig. 4E; *p* = 10^−322^, *n* = 2118).”

Additionally, this Article contained a typographical error in the Acknowledgements section.

“Patchanat Klaihmon”

now reads:

“Phatchanat Klaihmon”

These errors have now been corrected in the PDF and HTML versions of the Article.

