# Supplementary material for: Selective Tropism of Dengue Virus for Human Glycoprotein Ib
Source: Sci Rep. 2018 Feb 9;8:2688. doi: 10.1038/s41598-018-20914-z (PMC5807543; doi:10.1038/s41598-018-20914-z)
Supplement: Supplementary file 1 — Supplementary Information [file 41598_2018_20914_MOESM1_ESM.docx]

Supplementary Materials for

**Selective Tropism of Dengue Virus for Human Glycoprotein Ib**

Nattapol Attatippaholkun*, Nont Kosaisawe, Yaowalak U-Pratya, Panthipa Supraditaporn, Chanchao Lorthongpanich, Kovit Pattanapanyasat, Surapol Issaragrisil*

*correspondence to: surapolsi@gmail.com or nicsnattapol@gmail.com

**
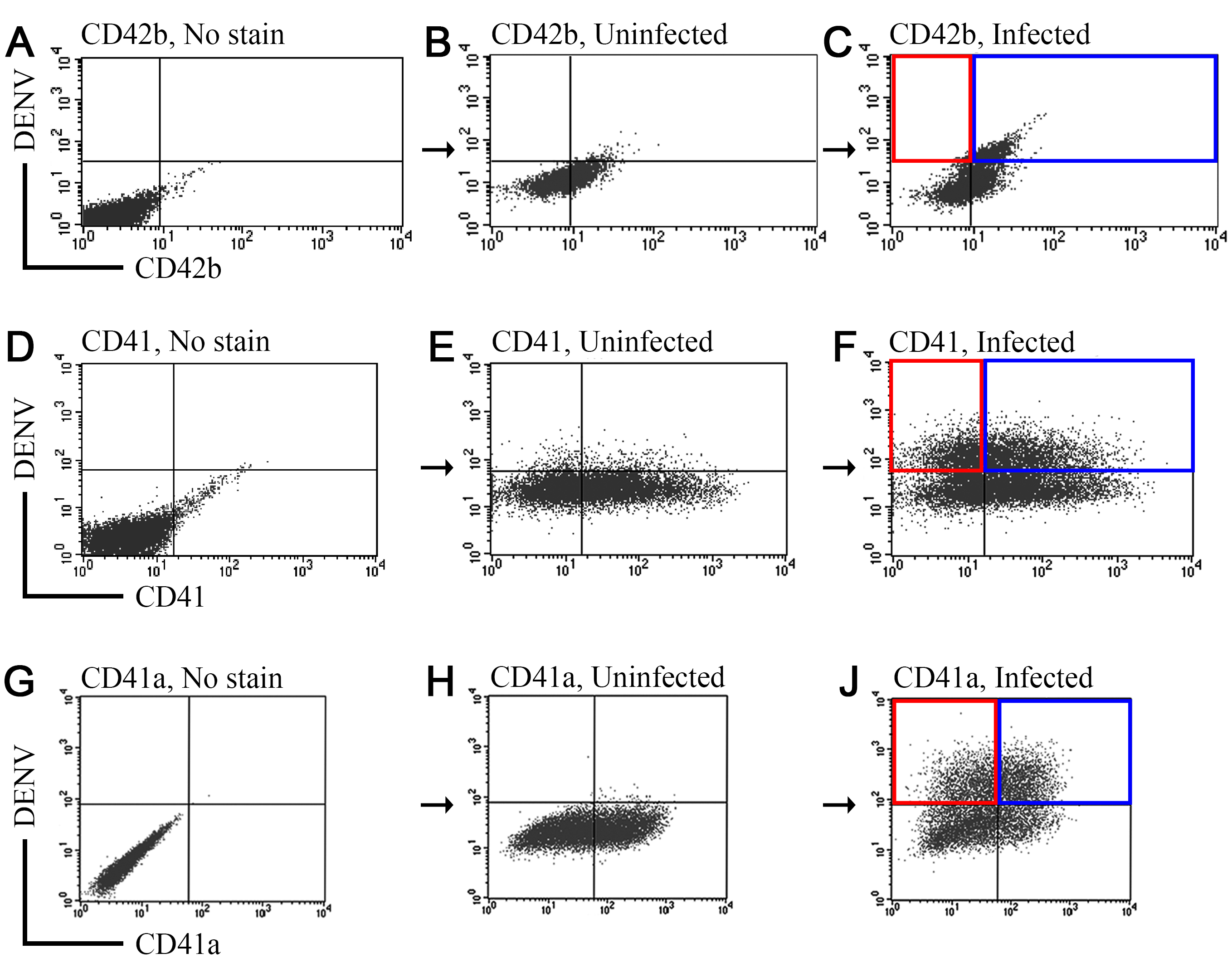
**

**Fig. S1. Defining the quadrant for analyzing surface platelet receptors and intracellular dengue virus in the tropism analysis.** The cutting points of cells with or without expressing the platelet receptors were based on the no-stained cells (A, D, G). The cutting points of cells with or without infected by DENV were based on the stained uninfected cells (B, E, H). DENV-infected cells were then analyzed by those two points (C, F, J). The analysis of intracellular DENV and surface CD42b (A-C), surface CD41 (D-F) and surface CD41a (G-J).


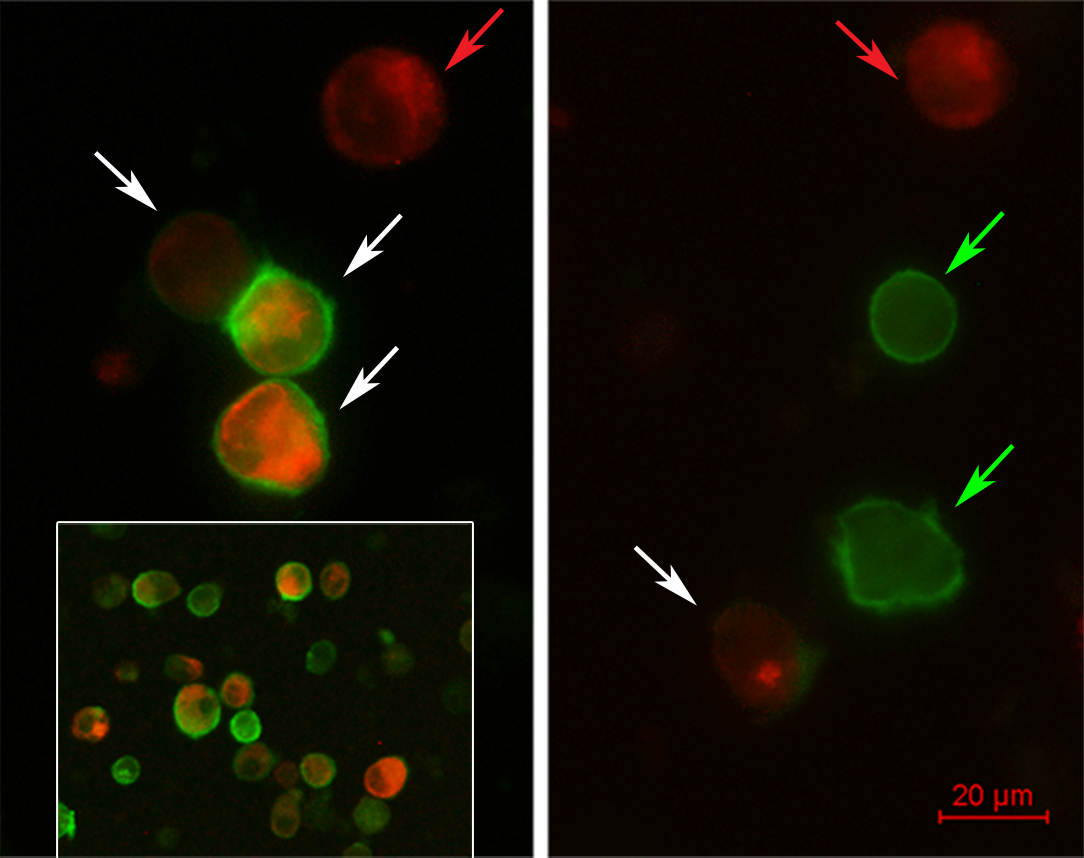


**Fig. S2. The binding locations of the antibodies by the immunostaining method used in this manuscript.** Our immunostaining method detected platelet receptors superficially and DENV intracellularly. MEG-01 cells were infected with DENV at MOI of 0 (Uninfected) and 0.5 (Infected) for 2 hrs and washed with PBS. The cultures were maintained in fresh medium for other 7 days before being double immunostained with FITC-anti CD41a antibody and PE-anti DENV antibody. White arrow are CD41a^+^DENV^+^ cells. Green arrow are CD41a^+^DENV^-^ cells. Red arrow are CD41a^-^DENV^+^ cells. Green is surface CD41a. Red is intracellular DENV.

**
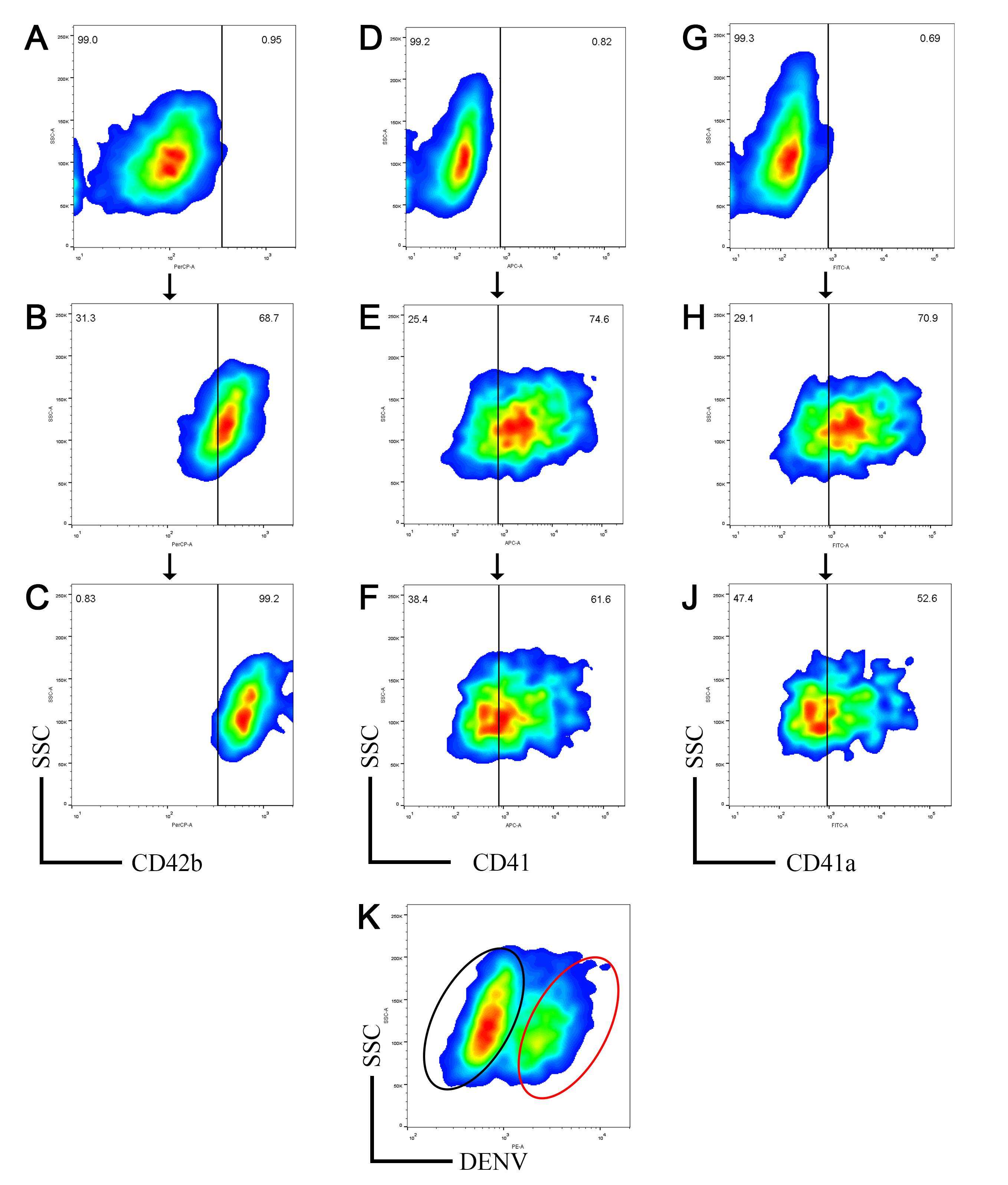
**

**Fig. S3. Defining the cutting point of cells with or without expressing platelet receptors in the specificity analysis.** The cutting points of cells with or without expressing the platelet receptors were based on the no-stained cells (A, D, G). The DENV^-^ or DENV^+^ cells were gated and further plotted based on two different subpopulations in the plot of SSC and DENV (K; black eclipse = DENV^-^ cells, red eclipse = DENV^+^ cells). DENV^-^ cells (B, E. H) and DENV^+^ cells (C, F, J) were further characterized by platelet receptors. The specificity analysis of DENV and CD42b (A-C), CD41 (D-F), CD41a (G-J).


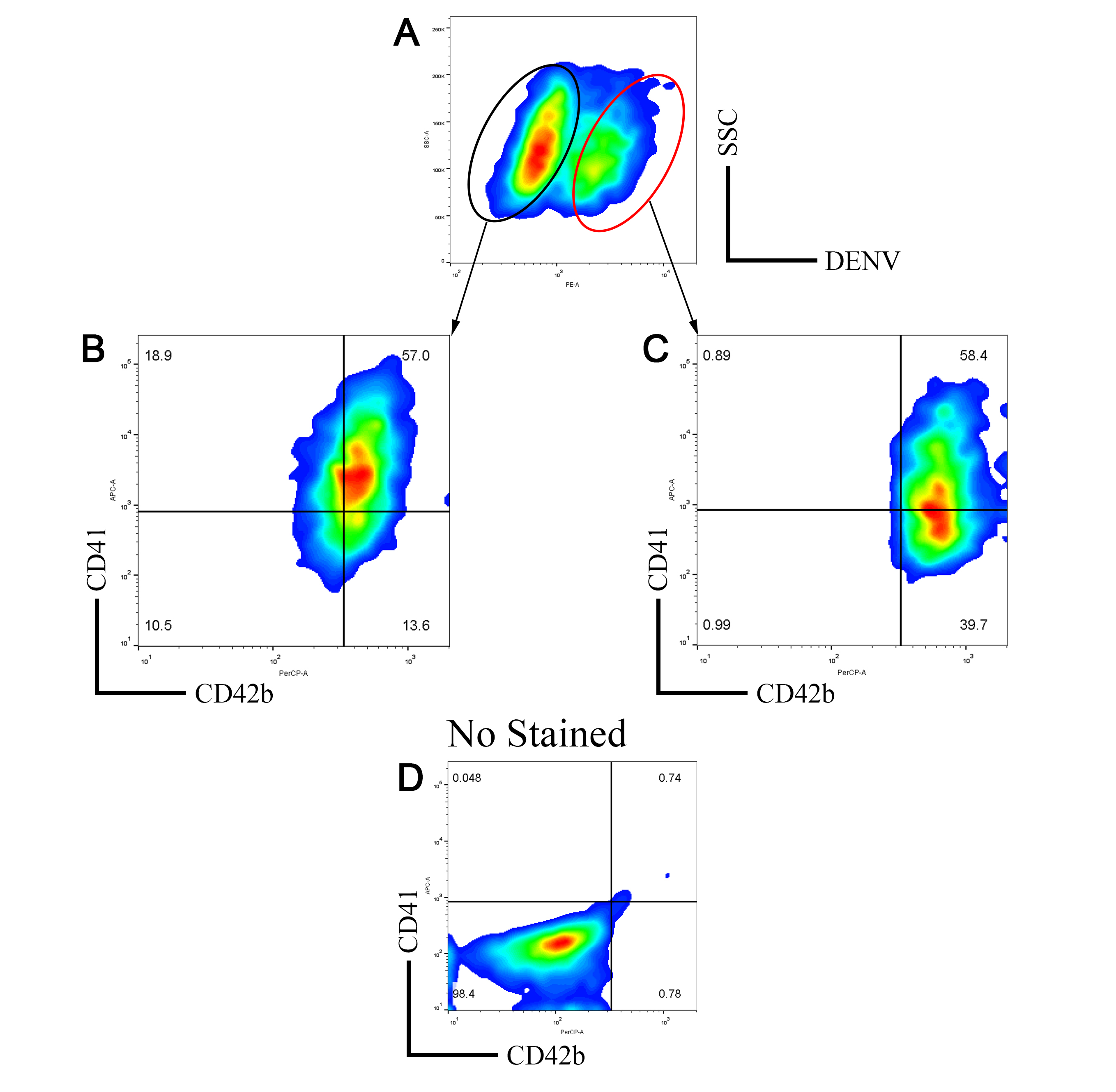


**Fig. S4. Two-dimensional analysis of triple immunostained DENV-infected cells in the requirement analysis.** The stained cells were firstly gated into two subpopulations as the following; DENV^-^ cells in black eclipse and DENV^+^ cells in red eclipse. The gated cells were further characterized with CD41 and CD42b (B; DENV^-^, C; DENV^+^). The cutting point of cells with or without expressing platelet receptor were based on no-stained cells (D).

**
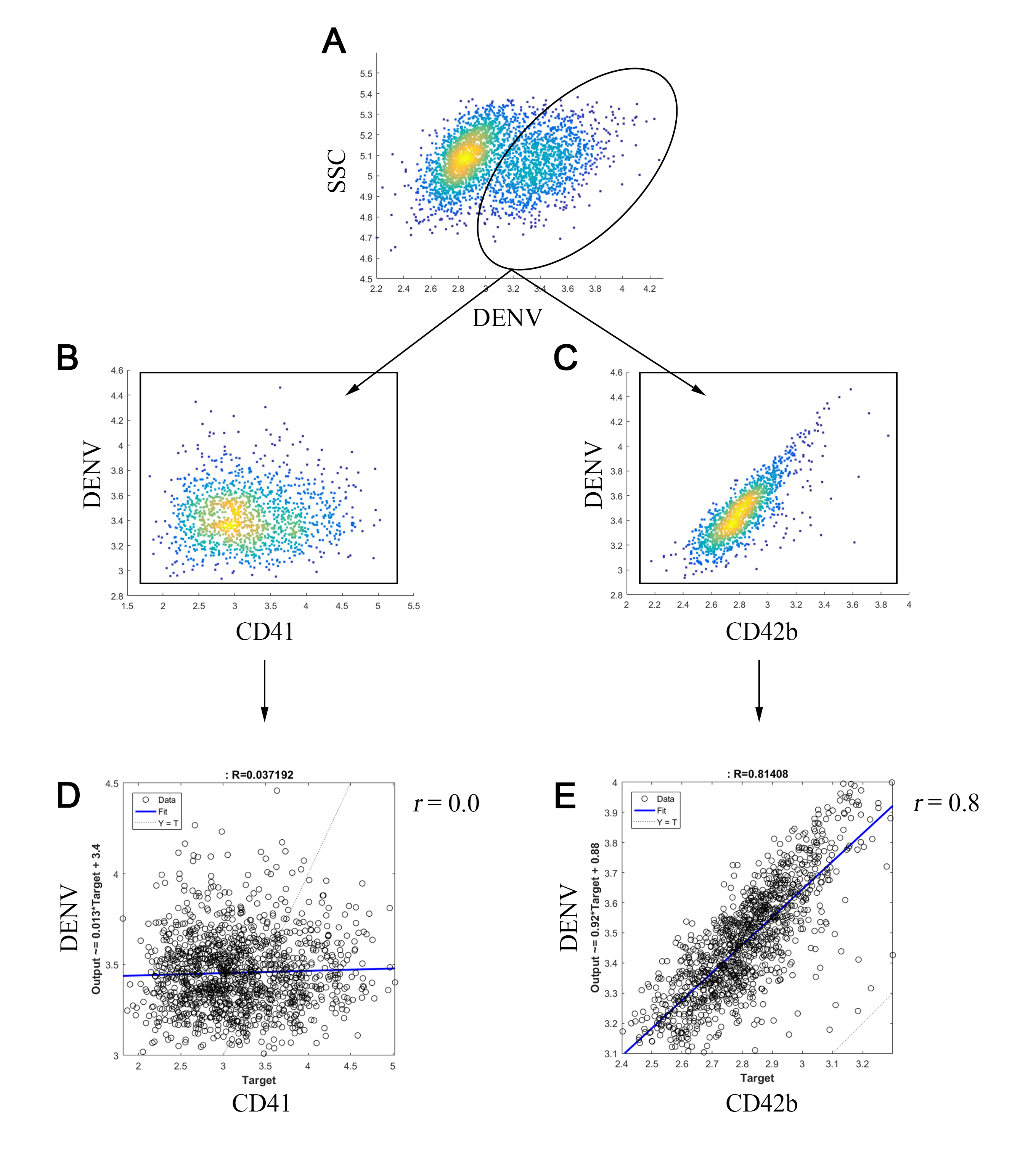
**

**Fig. S5. Single-cell correlation analysis before being excluded the non-specific signal.** Intracellular DENV^+^ cells were firstly gated (A; black eclipse) and then plotted in two-dimension of intracellular DENV and surface platelet receptors (B; CD42b, C; CD41). All the cells were gated (black square) for single-cell correlation analysis. (D) The analysis of surface CD41 and intracellular DENV (E) The analysis of surface CD42b and intracellular DENV. *r* = Pearson correlation coefficient.


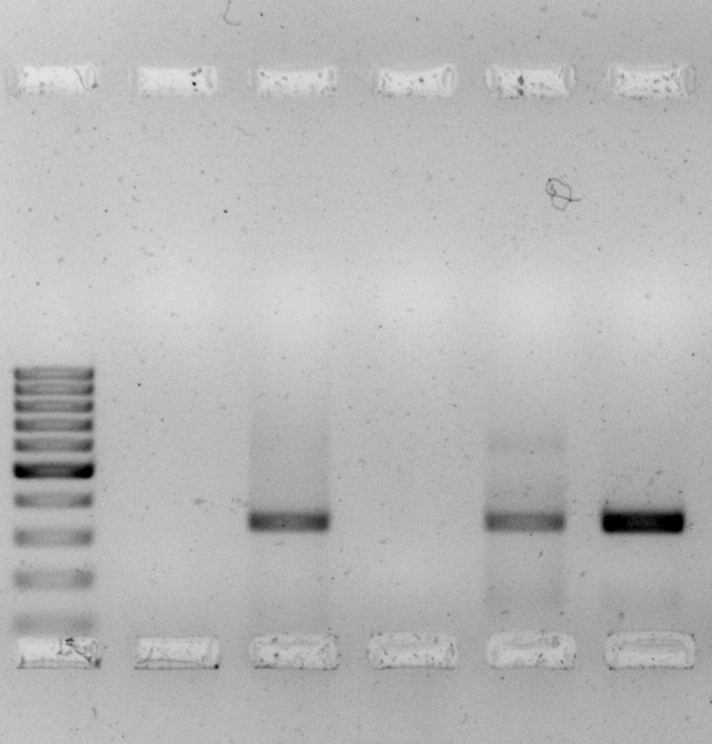

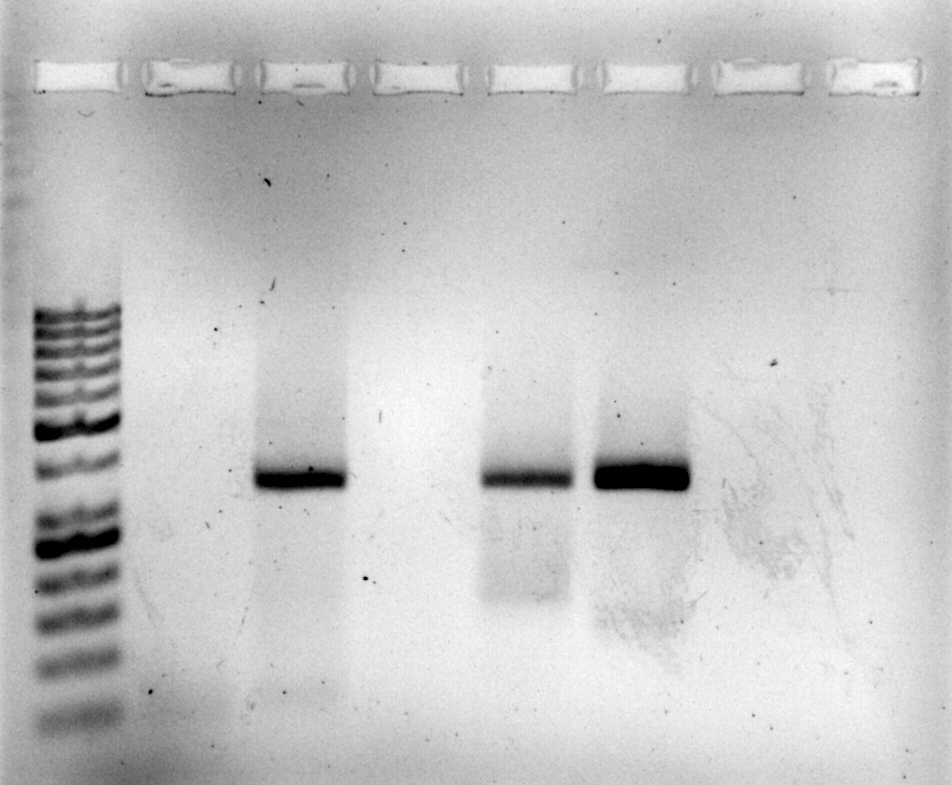


**Fig. S6. Full-length gels of figure 5A.**
